# kb_DRAM: annotation and metabolic profiling of genomes with DRAM in KBase

**DOI:** 10.1093/bioinformatics/btad110

**Published:** 2023-03-01

**Authors:** Michael Shaffer, Mikayla A Borton, Ben Bolduc, José P Faria, Rory M Flynn, Parsa Ghadermazi, Janaka N Edirisinghe, Elisha M Wood-Charlson, Christopher S Miller, Siu Hung Joshua Chan, Matthew B Sullivan, Christopher S Henry, Kelly C Wrighton

**Affiliations:** Colorado State University, Fort Collins, CO, USA; Colorado State University, Fort Collins, CO, USA; The Ohio State University, Columbus, OH, USA; Argonne National Laboratory, Lemont, IL, USA; Colorado State University, Fort Collins, CO, USA; Colorado State University, Fort Collins, CO, USA; Argonne National Laboratory, Lemont, IL, USA; Lawrence Berkeley National Laboratory, Berkeley, CA, USA; University of Colorado Denver, Denver, CO, USA; Colorado State University, Fort Collins, CO, USA; The Ohio State University, Columbus, OH, USA; Argonne National Laboratory, Lemont, IL, USA; Colorado State University, Fort Collins, CO, USA

## Abstract

Microbial genome annotation is the process of identifying structural and functional elements in DNA sequences and subsequently attaching biological information to those elements. DRAM is a tool developed to annotate bacterial, archaeal, and viral genomes derived from pure cultures or metagenomes. DRAM goes beyond traditional annotation tools by distilling multiple gene annotations to genome level summaries of functional potential. Despite these benefits, a downside of DRAM is the requirement of large computational resources, which limits its accessibility. Further, it did not integrate with downstream metabolic modeling tools that require genome annotation. To alleviate these constraints, DRAM and the viral counterpart, DRAM-v, are now available and integrated with the freely accessible KBase cyberinfrastructure. With kb_DRAM users can generate DRAM annotations and functional summaries from microbial or viral genomes in a point-and-click interface, as well as generate genome-scale metabolic models from DRAM annotations.

**Availability and implementation:**

For kb_DRAM users, the kb_DRAM apps on KBase can be found in the catalog at https://narrative.kbase.us/#catalog/modules/kb_DRAM. For kb_DRAM users, a tutorial workflow with all documentation is available at https://narrative.kbase.us/narrative/129480. For kb_DRAM developers, software is available at https://github.com/shafferm/kb_DRAM.

## 1 Introduction

Genome annotation is gene prediction followed by the assignment of biological function to genes. Protein coding sequences are commonly assigned function via homology searches to protein databases which contain sequences with assigned or inferred functional content. A number of genome annotators targeting microbial genomes have been developed (Aziz et al. 2008; Seemann 2014; Tanizawa et al. 2018; Dong and Strous 2019; Zhou et al. 2020). We previously developed DRAM, a genome annotation tool which allows the user to compile annotations from multiple functionally divergent protein databases at one time, then synthesizes this content into functional profiles for each genome (Shaffer et al. 2020). This allows the user to rapidly understand the collection of biologically encoded functions in a set of microbial genomes.

DRAM is limited by the high computational requirements of rapidly searching against large protein databases. It requires a minimum of 128 GB of RAM to set up and 64 GB of RAM to annotate. Thus, the use of DRAM is currently limited to those with access to large compute servers. Recently, cyberinfrastructure platforms have been built that provide access to computing resources as well as point-and-click interfaces to software that would usually require command-line access (Merchant et al. 2016; Afgan et al. 2018; Arkin et al. 2018).

Genome-scale metabolic models (GEMs) are representations of the metabolic reactions that occur within a bacterial cell. These reactions can be predicted from genome annotations, but many bacterial genome annotators do not generate output that is easily integrated into modeling frameworks. Additionally, recent research has shown value in combining annotations to improve genome function coverage (Griesemer et al. 2018), making frameworks with interoperable support of genome annotators and GEM construction more valuable.

We have built a DRAM KBase module (kb_DRAM). Here we show that using kb_DRAM, anyone with access to the KBase cyberinfrastructure (Arkin et al. 2018) can annotate microbial genomes with DRAM, distill these annotations into visualizations of predicted genomic functions, and use the annotations to build GEMs. Also, using bacterial genomes derived from phylogenetically distinct lineages, we show value added of including kb_DRAM annotation alongside an established KBase annotator, RAST (Aziz et al. 2008).

## 2 kb_DRAM

KBase is a cyberinfrastructure platform which allows users to use common bioinformatics tools to analyze public data or a user can upload their own. Within KBase users can process microbial genomics and metagenomics data from raw reads to assemblies and bins or imported data from other sources. kb_DRAM is a plugin that provides three KBase apps. These can (i) annotate microbial DNA sequences from assemblies, isolate genomes or metagenome-assembled genomes (KBase assembly objects), (ii) annotate predicted coding sequences from microbial genomes (KBase genome objects), or (iii) annotate viral genomes identified from metagenomes using DRAM-v.

The kb_DRAM apps use the same databases as the default DRAM installation [KOfam (Aramaki et al. 2020), dbCAN2 (Zhang et al. 2018), PFAM (El-Gebali et al. 2019), and MERPOS (Rawlings et al. 2018)] to annotate predicted microbial protein-coding genes as well as barrnap (https://github.com/tseemann/barrnap) for rRNA identification and tRNA-scanSE (Chan and Lowe 2019) for tRNA detection. Like DRAM, kb_DRAM generates and summarizes gene annotations across genomes into three levels of refinement: (i) Raw, (ii) Distillate, and (iii) Product (Supplementary Information). The raw is a synthesized annotation of all genes in a dataset across multiple databases, the distillate assigns many of these genes to specific functional categories, and the product visualizes the presence of key functional genes across genomes. Notably, the product is an interactive heatmap shown in the KBase browser that enables users to visually profile the functional potential of input genomes or metagenomes. All files generated by kb_DRAM (e.g. raw annotations, distillate, and genome completion) are available to download so users can understand their genomes more deeply. kb_DRAM apps also generate annotated KBase genome objects, which can be used in downstream analyses in KBase including building GEMs, not previously possible in DRAM alone. Each output from kb_DRAM is described in detail in the Supplementary Information.

DRAM-v is designed to annotate viral genomes that are identified using VirSorter (Roux et al. 2015). DRAM-v uses the same functional databases as DRAM with the addition of RefSeq viral. To annotate with DRAM-v within KBase users can start with metagenomic assemblies and identify potential viral contigs from metagenomes using the VirSorter app in KBase. The output of the VirSorter app is then passed to the DRAM-v app for auxiliary metabolic gene (AMG) annotation. The DRAM-v app shows the interactive product heatmap, which highlights potential AMGs identified in the dataset along with confidence scores for each and allows the user to download all other DRAM-v files.

## 3 DRAM annotations of microbial genomes can generate quality GEMs in KBase

**Figure 1:**
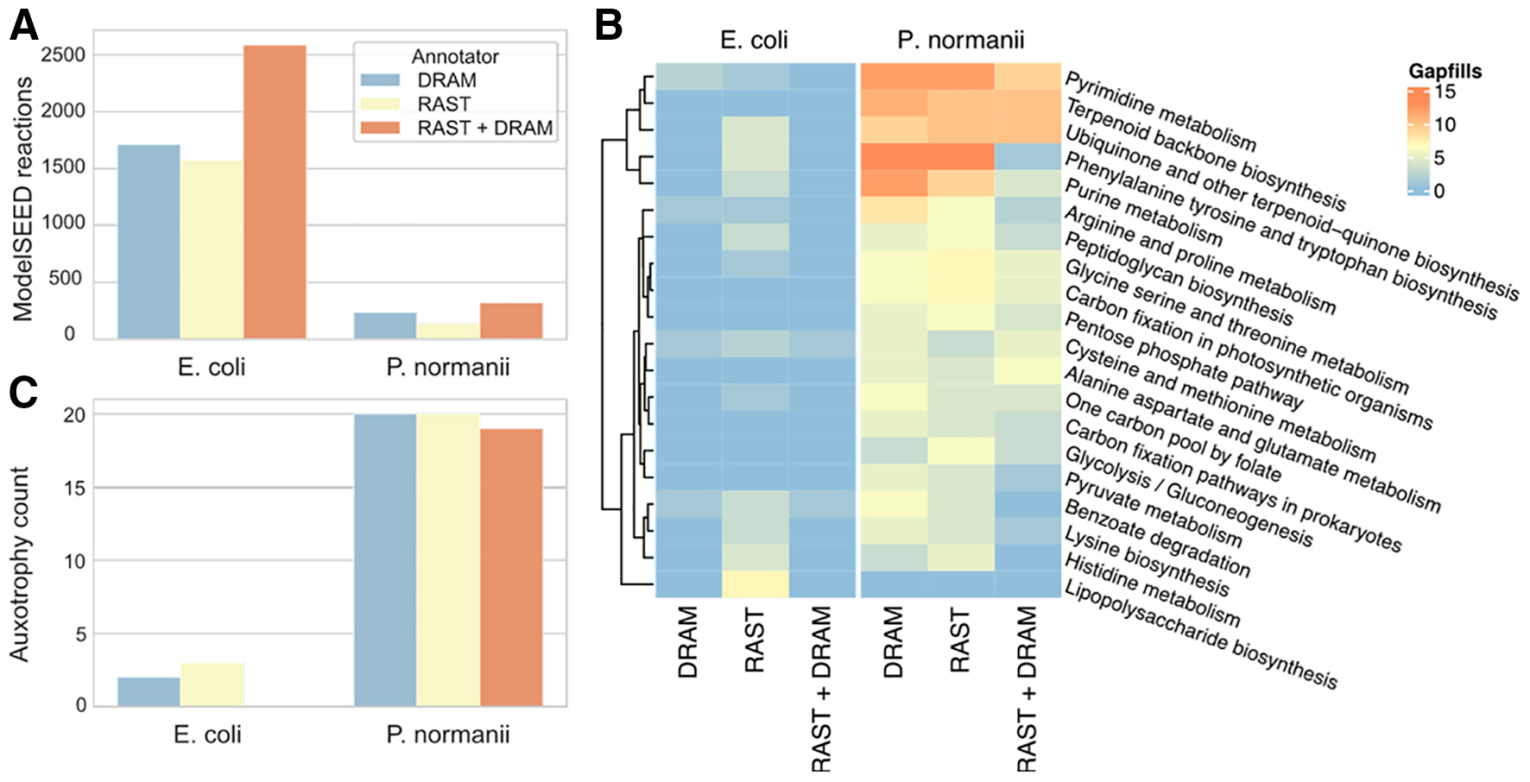
Annotation and modeling performance on three genomes. (A) Number of modelSEED reactions assigned by each tool on each genome. (B) Heatmap showing number of gap filled reactions required for growth on glucose minimal media per pathway. Only pathways with ≥5 gap fills required by at least one annotation set shown. (C) Number of auxotrophies predicted present based on annotations from each tool for each genome

To demonstrate DRAM in KBase and show its compatibility with downstream applications, we annotated two genomes with the RAST and DRAM apps. *Escherichia coli* strain K-12 was chosen as a well-characterized bacterial genome, while *Paceibacter normanii* AAA255-P19, a member of the candidate phyla radiation, represents a genome obtained from uncultivated microbes through metagenomics with limited functional curation (Castelle et al. 2018). Full workflows with these data are available on KBase (*E.coli*: https://kbase.us/n/103341/23/, *P.normanii*: https://kbase.us/n/128174/5/), and Supplementary Fig. S1 displays the methods workflow for this analysis in KBase. For both genomes, when comparing the outputs of DRAM to RAST, DRAM annotations yielded more ModelSEED reactions, a measure of how many unique gene annotations could be converted to metabolic reactions (Fig. 1A, Supplementary Table S1). As expected, due to the depth of study of *E.coli*, we obtained more reaction-specific annotations for *E.coli* and much fewer for *P.normanii*. Ultimately, as others have shown (Griesemer et al. 2018), there was value in using more than one annotator, as merged DRAM + RAST annotations yielded 1.5× and 1.4× more reactions than DRAM and 1.6× and 2.3× for RAST in *E.coli* and *P.normanii*, respectively.

Next, we constructed GEMs using the RAST, DRAM, and DRAM + RAST annotations and GEMs were gap filled using glucose minimal media to bridge gaps in metabolic pathway reconstruction leading to biomass production (see Supplementary Information). The kb_DRAM app represents a significant advance, as DRAM annotations can be directly integrated into GEMs, a capability not previously available in DRAM.

*Escherichia coli* RAST had more reactions (*n* = 1681) in the model than DRAM but fewer in *P.normanii* (*n* = 488). In both cases, DRAM + RAST outperformed each annotator alone (Supplementary Table S1). We failed to find a clear pattern of better performance by DRAM or RAST in any particular metabolic pathway (Fig. 1B). Subsequently, the GEMs were characterized to predict auxotrophies. In all genomes the merged annotation model showed the least number of auxotrophies (Fig. 1C). Interestingly for the *E.coli* model both RAST and DRAM predicted auxotrophies, while merging the annotations removed these auxotrophies, yielding a final GEM more consistent with expected experimental evidence (Tao et al. 1999). We note that a large number of auxotrophies are still predicted for *P.normanii*, even when merging annotations. This finding may be biological, reflecting the symbiotic lifestyle predicted for members of this species, as many of these reactions could be provided by the host (He et al. 2021). However, there is no experimental data for *P.normanii* to validate this inference at this time.

## 4 Conclusion

Here, we present a KBase module with apps for running DRAM and DRAM-v. This resource enables computationally intensive genome annotation by broader audiences. We highlight that both DRAM-v and DRAM are integrated into the KBase cyberinfrastructure with the ability to ingest data from and pass data to other KBase applications. We show that DRAM can be applied to generate gene annotations from phylogenetically distinct genomes derived from pure cultures and metagenomics. Using *E.coli*, we demonstrated the addition of DRAM annotations yielded a GEM that was consistent with experimental evidence. Thus, the kb_DRAM app will enhance user analyses of genome function beyond DRAM, enabling seamless integration of DRAM gene annotations into modeling frameworks.

## Supplementary Material

btad110_Supplementary_Data
