# Correction to: kb_DRAM: annotation and metabolic profiling of genomes with DRAM in KBase

**DOI:** 10.1093/bioinformatics/btae325

**Published:** 2024-05-25

This is a correction to: Michael Shaffer, Mikayla A Borton, Ben Bolduc, José P Faria, Rory M Flynn, Parsa Ghadermazi, Janaka N Edirisinghe, Elisha M Wood-Charlson, Christopher S Miller, Siu Hung Joshua Chan, Matthew B Sullivan, Christopher S Henry, Kelly C Wrighton, kb_DRAM: annotation and metabolic profiling of genomes with DRAM in KBase, Bioinformatics, Volume 39, Issue 4, April 2023, btad110, https://doi.org/10.1093/bioinformatics/btad110

In the originally published version of this article, the acknowledgement of the grant from the National Institutes of Health [R01AI143288] was inadvertently omitted.

The article’s Funding section has been corrected to include this information.

