# Supplementary material for: kb_DRAM: annotation and metabolic profiling of genomes with DRAM in KBase
Source: Bioinformatics. 2023 Mar 1;39(4):btad110. doi: 10.1093/bioinformatics/btad110 (PMC10068739; doi:10.1093/bioinformatics/btad110)
Supplement: btad110_Supplementary_Data [file btad110_supplementary_data.zip › SOM_v2.pdf]

Supplementary Information for "**kb_DRAM: Annotating and functional profiling of genomes with DRAM in KBase"**

Supplementary Methods

Genome Scale Metabolic Modeling in KBase. To evaluate GEMs built from DRAM annotations we annotated two genomes using kb_DRAM (version 0.0.2) and RAST, the primary annotation system on KBase (version b04268c) (Aziz *et al*., 2008). The genomes selected were *Escherichia coli* str. K-12 substr. MG1655 (NC_000913) and *Paceibacter normanii* SCGC AAA255-P19 (GCA_000398025.1). For both annotators, default parameters in DRAM and RAST were used. The Merge Metabolic Annotations app (version 1.2.11) (Griesemer *et al.*, 2018) was used to merge the KO and EC annotations from DRAM (called the DRAM annotations here) and these were merged with the RAST annotations using the same app to generate the DRAM + RAST annotations. Breifly, merging annotations is necessary because DRAM annotates using EC numbers and KO terms, while RAST annotates using SEED functional roles. All these terms by themselves are difficult to reconcile, but KBase maintains a curated set of mappings between all these terms and the ModelSEED biochemistry database (https://modelseed.org/biochem). When merging DRAM and RAST annotations, we translate all the metabolic annotations to ModelSEED reaction IDs using this mapping, then these are the reactions integrated into the metabolic models.

Models were generated from all three sets of annotations (DRAM, RAST, DRAM + RAST) with the Build Metabolic Model app (version 2.0.0) with internal annotations not used and nontemplate reactions included (cite). Gap filling was done using glucose minimal media. Model quality was assessed using the MEMOTE app (version d8afc8c) (Lieven *et al.,* 2020). Auxotrophies and ATP production by the models were predicted using Run Model Characterization (version 15fabbd) (Arkin *et al.*, 2018; Edirisinghe, et al., 2016).

Gapfilling GEMs in KBase. Draft metabolic models usually have missing reactions due to incomplete or incorrect functional genome annotations. As a result, these models are unable to generate biomass on media where the organism typically is capable of growing. Gap filling algorithms can be used to overcome this problem. These algorithms tentatively bridge gaps in metabolic pathways by identifying

the minimal number of biochemical reactions to add to the draft metabolic model, thereby enabling it to produce biomass in a specified media. Gap filling in KBase can be done at the metabolic reconstruction step or using the Build Metabolic Model App. Gap filling using either method in KBase is achieved by one of two ways: (i) relaxing reversibility constraints on the models reactions or (ii) adding new reactions to the existing model. In this gapfilling process, the model is augmented to include all (i.e., about 13,000) biochemical reactions contained in the ModelSEED database. The database consists of reactions from KEGG, MetaCyc, EcoCyc, plant BioCyc, Plant Metabolic Networks, and Gramene. During the gap filling process, all reactions determined to be thermodynamically reversible are adjusted to be reversible in the gap filled metabolic model. Finally, flux balance analysis (FBA) is performed to generate a flux profile that prioritizes the production of biomass while minimizing the flux through all reactions and reaction directions that were added in the gap filling process.

kb_DRAM module descriptions

kb_DRAM utilizes three different input file types:

(1) For the annotation of microbial DNA sequences from assemblies, isolate genomes, or metagenome assembled genomes, use KBase assembly objects

(2) For the annotation of predicted coding sequences from microbial genomes, use KBase genome objects

(3) For the annotation of viral genomes identified form metagenomes using DRAM-v, use Ishock ID from the KBase VirSorter Summary

Annotate and Distill Assemblies with DRAM DRAM will predict coding sequences and annotate input microbial DNA sequences from assemblies, isolate genomes or MAGs (KBase assembly objects) and provide genome metabolic summaries. DRAM is most useful for annotating genome sets, where the DRAM product can be used to compare the metabolic profiles of each genome. DRAM for assemblies works by predicting coding sequences and annotating all genes given in a KBase Assembly object with a set of databases curated to the task.

Annotate and Distill Genomes with DRAM DRAM will annotate input predicted coding sequences from microbial genomes (KBase genome objects) and provide genome metabolic summaries. DRAM is most useful for annotating genome sets, where the DRAM product can be used to compare the metabolic profiles of each genome. DRAM for genomes works by annotating all genes given in a KBase Genome object with a set of databases curated to the task.

Annotate and Distill Viral Assemblies with DRAM-v, or DRAM for vMAGs, will annotate vMAGs and predict potential auxiliary metabolic genes, through a set of rules defined in https://academic.oup.com/nar/article/48/16/8883/5884738. DRAM for vMAGs works by annotating viral genomes with a set of databases curated to the task, and integrating additional input from Virsorter. Note that you must start with a metagenomic assembly object and run the VirSorter KBase app. DRAM-v is run using the viral genome files along with the lshock ID from the KBase VirSorter Summary.

kb_DRAM output file descriptions

kb_DRAM for MAGs and metagenomes, run "Annotate and Distill Assemblies with DRAM"

(1) *Raw* (found in the "Files" dialog at completion)

- Tab separated file (.tsv) with all the annotations from Pfam (1), KOfam (2), UniProt90 (3), VOGDB (http://vogdb.org/), dbCAN (4), and MEROPS (5) databases for all genes in all input genomes
- GenBank (.gbk) files for each genome, compiled into a tar.gz
- General feature format (GFF) (.gff) file of all annotations across genomes
- FASTA format file (.fna) of each open reading frame nucleotide sequence and best ranked annotation (see Annotation ranks section)
- FASTA format file (.faa) of each translated open reading frame amino acid sequence and best ranked annotation KEGG (2) annotation
- Tab separated file (.tsv) with the count and position of the detected tRNAs
- Tab separated file (.tsv) with the count and position of the detected rRNAs

(2) *Distillate* (found in the "Files" dialog at completion)

- Tab separated file (.tsv) with genome statistics for all input genomes including all mandatory genome quality statistics required by recently defined MIMAG standards (6)
- Microsoft Excel spreadsheet (.xlsx) with metabolism summary of all input genomes, which gives gene counts of functional and structural genes across a wide variety of metabolisms

(3) *Product*

- HTML (.html) file containing an interactive heatmap showing coverage of pathways, the coverage of electron transport chain components, and the presence of selected metabolic functions (Found in the "Report", and in the "Links" dialog at completion)
- Tab separated file (.tsv) with data from the html heatmap (found in the "Files" dialog at completion)

kb_DRAM for MAGs and metagenomes, run "Annotate and Distill Genomes with DRAM"

(1) *Raw* (found in the "Files" dialog at completion)

- Tab separated file (.tsv) with all the annotations from Pfam (1), KOfam (2), UniProt90 (3), VOGDB (http://vogdb.org/), dbCAN (4), and MEROPS (5) databases for all genes in all input genomes
- General feature format (GFF) (.gff) file of all annotations across genomes
- FASTA format file (.fna) of each open reading frame nucleotide sequence and best ranked annotation (see Annotation ranks section)
- FASTA format file (.faa) of each translated open reading frame amino acid sequence and best ranked annotation KEGG (2) annotation

(2) *Distillate* (found in the "Files" dialog at completion)

- Tab separated file (.tsv) with genome statistics for all input genomes including all mandatory genome quality statistics required by recently defined MIMAG standards (6)
- Microsoft Excel spreadsheet (.xlsx) with metabolism summary of all input genomes, which gives gene counts of functional and structural genes across a wide variety of metabolisms

(3) *Product*

- HTML (.html) file containing an interactive heatmap showing coverage of pathways, the coverage of electron transport chain components, and the presence of selected metabolic functions (Found in the "Report", and in the "Links" dialog at completion)
- Tab separated file (.tsv) with data from the html heatmap (found in the "Files" dialog at completion)

kb_DRAM for vMAGs , run "Annotate and Distill Viral Assemblies with DRAM"

(1) *Raw* (found in the "Files" dialog at completion)

- Tab separated file (.tsv) with all the annotations from PFAM (1), KEGG (2), Uniref90 (3), dbCAN (4), MEROPS (5), VOGDB (http://vogdb.org/), and the viral subset of RefSeq (7) for all genes
- Folder of GenBank files with annotations for each viral contig
- General feature format (.gff) file of all annotations across viral contigs
- FASTA format file (.fna) of each open reading frame nucleotide sequence and best ranked annotation
- FASTA format file (.faa) of each translated open reading frame amino acid sequence and best ranked annotation
- Tab separated file (.tsv) with the count and position of the detected tRNAs

(2) *Distillate* (found in the “Files” dialog at completion)

- Tab separated file (.tsv) with viral contig statistics for all input viral contigs including some statistics required by recently defined MIUVIG standards (8)
- Tab separated file (.tsv) of auxiliary metabolic genes (AMG) summary from all input viral contigs, which lists putative AMGs with annotation, auxiliary scores, and other flags outlined in main text Figure 6ab.

(3) *Product*

- HTML (.html) file containing an interactive heatmap showing all viruses with a putative AMG and the AMG metabolism category, with number of AMGs on each contig noted (Found in the “Report”, and in the “Links” dialog at completion)
- Tab separated file (.tsv) with corresponding AMGs from the html heatmap (found in the “Files” dialog at completion)

Supplementary Files

Table 1: Genome scale metabolic model comparison of different annotation methods on two genomes, with the ModelSEED rxns referring to the total number of unique annotation terms recovered from each annotation and model reactions referring to the number of reactions in the model.

File 1: Workflow diagram of methods from annotations to models in KBase.

Supplementary References

Arkin, Adam P., et al. "KBase: the United States department of energy systems biology knowledgebase." *Nature biotechnology* 36.7 (2018): 566-569.

Aziz, Ramy K., et al. "The RAST Server: rapid annotations using subsystems technology." *BMC genomics* 9.1 (2008): 1-15.

Edirisinghe, Janaka N., et al. "Modeling central metabolism and energy biosynthesis across microbial life." *Bmc Genomics* 17.1 (2016): 1-11.

Griesemer, Marc, et al. "Combining multiple functional annotation tools increases coverage of metabolic annotation." *BMC genomics* 19.1 (2018): 1-11.

Lieven, Christian, et al. "MEMOTE for standardized genome-scale metabolic model testing." *Nature biotechnology* 38.3 (2020): 272-276.
