# Supplementary material for: kb_DRAM: annotation and metabolic profiling of genomes with DRAM in KBase
Source: Bioinformatics. 2023 Mar 1;39(4):btad110. doi: 10.1093/bioinformatics/btad110 (PMC10068739; doi:10.1093/bioinformatics/btad110)

Visualization of a Typical KBase Narrative Using DRAM

This Narrative is Available Here

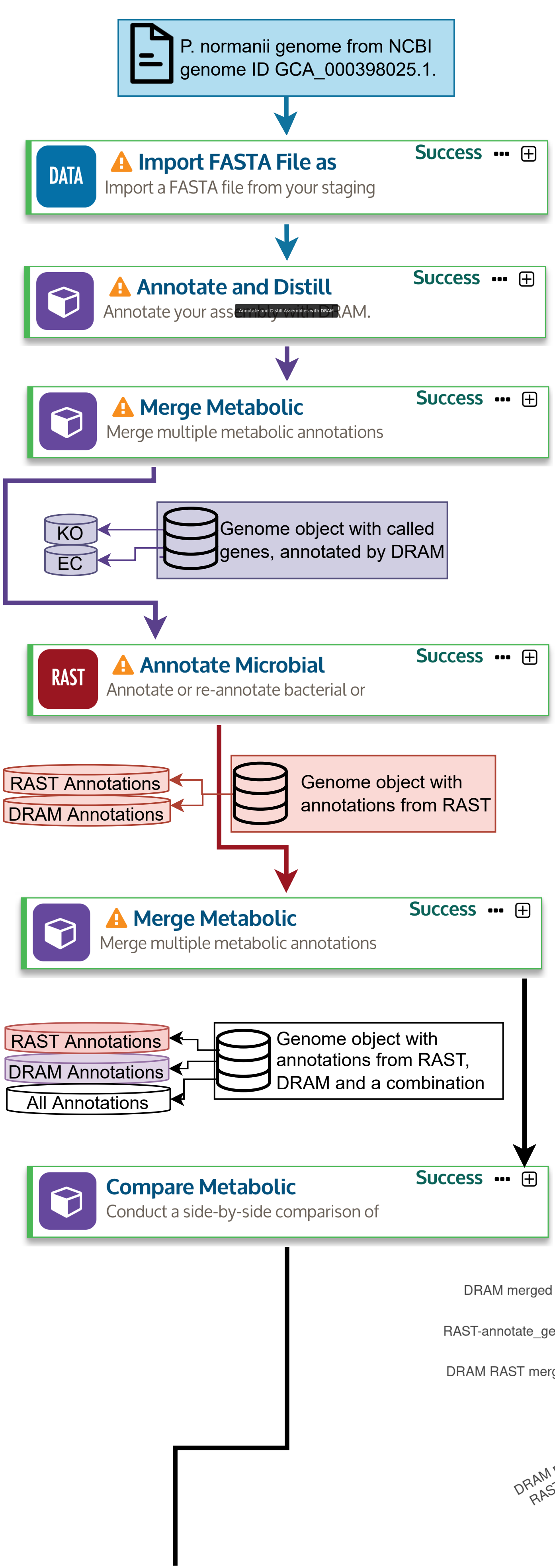

Load Your Data

Before users can use kb_DRAM they need data in KBase. Using the GUI users can upload a fasta file then import that fasta as a Genome Assembly. Here we are using a metagenome assembled genome fasta for P. normanii from NCBI genome ID GCA_000398025.1.

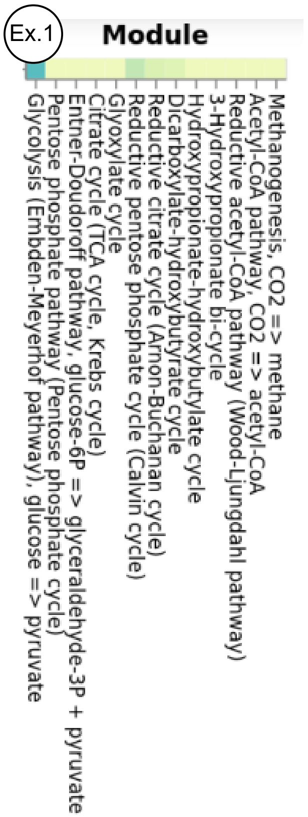

Call Genes with DRAM and Merge Them

Use "Annotate and Distill Assemblies with DRAM" to call genes. DRAM generates 2 sets of annotations, called annotation events, KOs and ECs . The KOs come from annotation with KOfam and EC annotations from KOfam and DBCAN2. The visual output of this is the DRAM distillate, partly shown in Ex.1.

As a formatting step, merge these two annotation events, resulting in a Genome Object with multiple annotations contained within.

Annotate Genes with RAST

Called genes, output from DRAM, can also be annotated with RAST. This makes a new Genome object with separate RAST annotations event.

Merge Annotations Once More

Separate RAST and DRAM annotations, can be combined to get the full set. This results in genome objects, with all 3 annotation events combined, to be used for the rest of the steps.

Compare Annotations

KBase provides excellent tools to compare our three annotation events. Ex.2 and Ex.3 show 2 of the figures summarizing unique features by annotation events.

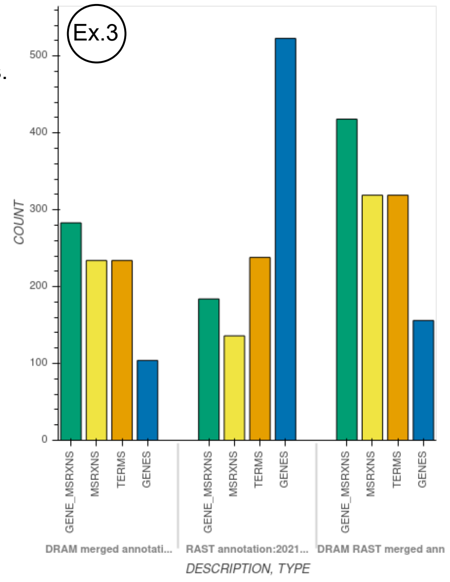

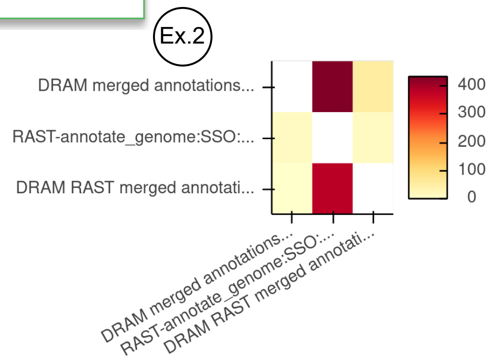

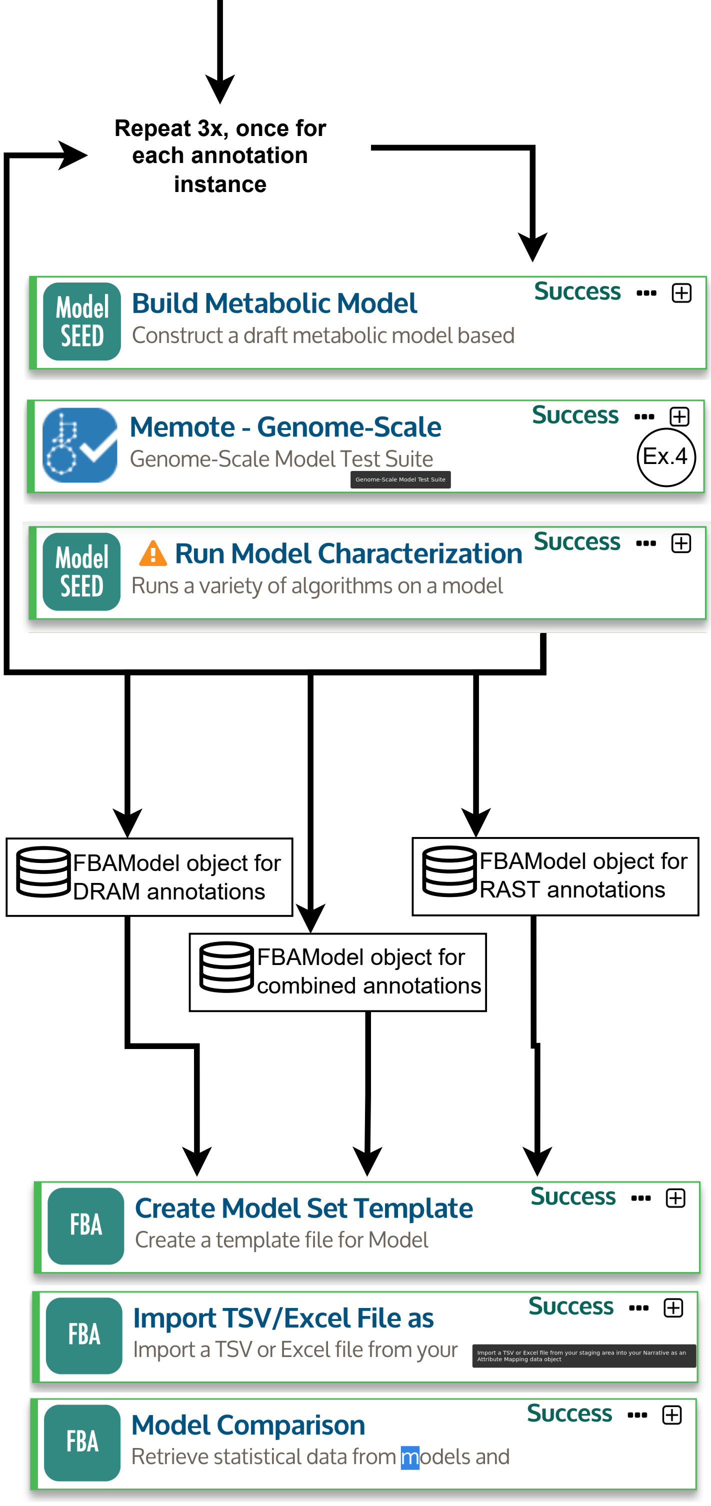

Build, Test, and Characterize GEMs

KBase offers an unparalleled set of tools to construct genome-scale metabolic models. Models are constructed based on the ModelSEED Pipeline, tested by Memote, and characterized in terms of quality, pathways, and auxotrophy. Ex.4 shows the output of just one part of the testing suite available.

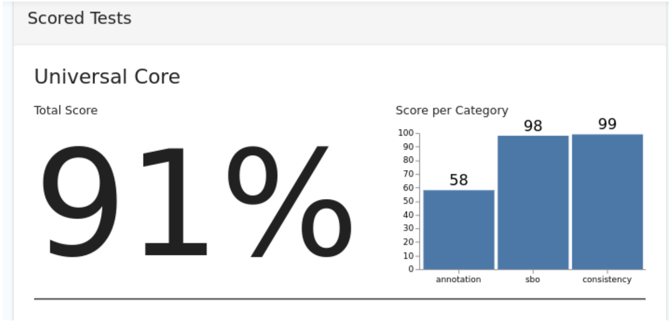

Compare all GEMs

Using the models created, users can use KBase model comparison tool to understand them. Users create a model comparison template and import it into the work space before running Model comparison to get a bevy of information, including the Pathway Comparison Heatmap in EX.5.

Ex.5

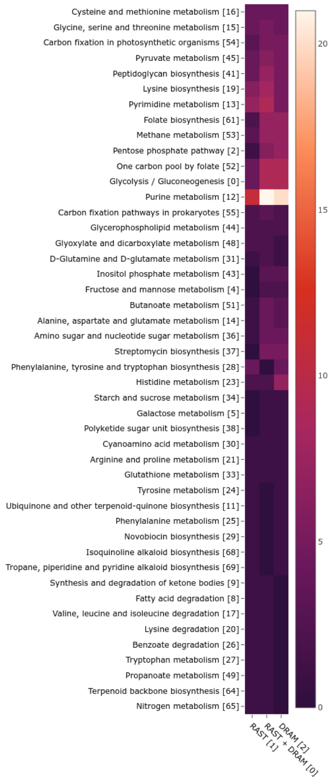

Supplement: btad110_Supplementary_Data [file btad110_supplementary_data.zip › SuppFile1.pdf]
